# Harnessing Knowledge from Plant Functional Genomics and Multi-Omics for Genetic Improvement

**DOI:** 10.3390/ijms241210347

**Published:** 2023-06-19

**Authors:** Yaqiong Wang, Jian Zeng, Guangxiao Yang, Yongfang Wan, Yin Li

**Affiliations:** 1 The Genetic Engineering International Cooperation Base of Chinese Ministry of Science and Technology, The Key Laboratory of Molecular Biophysics of Chinese Ministry of Education, College of Life Science and Technology, Huazhong University of Science & Technology, Wuhan 430074, China; wangyq@hust.edu.cn (Y.W.); ygx@hust.edu.cn (G.Y.); 2 Guangdong Provincial Key Laboratory of Utilization and Conservation of Food and Medicinal Resources in Northern Region, Henry Fok School of Biology and Agriculture, Shaoguan University, Shaoguan 512005, China; zengjian@sgu.edu.cn; 3 Sustainable Soils and Crops Department, Rothamsted Research Centre, Harpenden, Hertfordshire AL5 2JQ, UK

## 1. Introduction

Plant biology research has currently entered the post-genomics era with the advances in genomic technologies. The unprecedented development of genomics and application in crops is driving a new “green revolution” in agriculture. With the high-quality genomes of many important crops (for example, rice, wheat, maize, soybean, and vegetables), numerous genes controlling the agronomically important traits have been identified and the underlying mechanisms have been revealed.

Modern plant genomics can boost research progress in several aspects: (1) omics data provide a comprehensive exploration of a given gene family in a genome-wide, sometimes genus-wide manner; (2) high-quality genomes and large-scale sequencing data allow the identification of single nucleotide polymorphism (SNP) and structural variations, facilitating quantitative trait loci (QTL) or genome-wide association (GWAS) studies to rapidly identify genetic loci for agronomic traits; (3) combining with other omics, such as metabolomics, quantitative proteomics, epigenomics and epitranscriptomics, multi-omic approaches can gain new insights into a biological question even without an available reference genome.

In this context, the present Topic “Plant Functional Genomics and Crop Genetic Improvement” provides a forum for researchers to communicate their latest findings related to plant functional genomics and the corresponding applications in crop genetic improvement. We collaborate with several related journals, including Agronomy, Crops, International Journal of Molecular Sciences, Life and Plants, to cover such a broad-range theme. This Topic has collected a set of 51 papers (three reviews and 48 research articles) with a relatively low acceptance rate (~25%), covering several species including wheat (*Triticum aestivum* L.), rice (*Oryza sativa* L.), maize (*Zea mays* L.), soybean (*Glycine max* L.), cotton (*Gossypium hirsutum* L.), rapeseed (*Brassica napus* L.), tea plant (*Camellia sinensis* (L.) O. Kuntze), flowering Chinese cabbage (*Brassica campestris* L. ssp. *chinensis*) and many others. These studies fall into four themes of plant genetics and genomics: (1) Genome-wide characterization of gene families in plants; (2) Functional studies of genes regulating various traits in plants; (3) Dissecting important agronomic traits through population genetics; (4) Multi-omic analysis facilitates plant functional genomic and genetic improvement studies. This Editorial paper aims to showcase the Topic and discuss the related perspectives.

## 2. Genome-Wide Characterization of Gene Families in Plants

Genome-wide study of a gene family illustrates the phylogeny and sub-functionalization, combines multi-omic data sets, and depicts the contraction and expansion of the family, serving as a starting point for functional experiments for certain family members. In this topic, 12 genes of serine hydroxy-methyltransferase (*SHMT*), 227 genes of basic leucine zipper (*bZIP*) and 10 genes of catalases (*CATs*) were identified in wheat, and the members with potentially regulating plant growth and the responses to various abiotic stresses were explored in several studies [1,2,3]. Another study identified 75 members of the expansin (*EXP*) family in wild soybean (*Glycine soja*). In the identified 75 members of the expansin (*EXP*) family, overexpression of *GsEXPB1* increased the number of hairy root and tolerance to salt stress [4]. Sehrish et al. identified 122 SET domain genes (*SDGs*) genes in *B.napus* and characterized their evolution, structure and expression patterns in detail [5]. In several less-studied species, genome-wide characterization of the YABBY family in *Juglans regia* and *Juglans mandshurica* [6], glutathione S-transferase (*GST*) in pear (*Pyrus communis*) [7], and the *MADS-Box* transcription factor family in Safflower (*Carthamus tinctorius* L.) [8] have broadened our knowledge regarding their evolution and association with agronomic traits in the above-mentioned species.

Plant xyloglucan endo-transferases (XETs), are one group of the key factors that underlie plant cell wall dynamics and mechanistic. A comprehensive review of the functions, taxonomy, protein structures and catalytic mechanisms of XETs was presented by Hrmova et al. [9]. The broad specificity of plant XETs was explained and their roles in cell wall reorganization and reconstruction was emphasized.

Li et al. conducted a comparative analysis of the *SQUAMOSA-PROMOTER BINDING PROTEIN-LIKE* (SPL) gene family between rice and wheat and established the orthologous relationship between *TaSPLs* and *OsSPLs*. The authors further demonstrated the functional conservation of *TaSPL3* with OsSPL3 in the regulation of plant height, and flowering time by using transgenic rice [10].

## 3. Functional Studies of Genes Regulating Various Traits in Plants

Our topic hosts a large number of gene functional studies that involve the traits related to yield and production (e.g., plant architecture, yield, grain weight, and nutrient use efficiency), development and metabolism (e.g., fatty acid and flower color) to biotic and abiotic stress resistance (such as pathogen or weed resistance).

Crop plant architecture traits, consisting of plant height, tiller number, tiller angle, and panicle morphology, are important for grain yields. Wang et al. identified a new recessive semi-dwarf gene, *Rht-SN33d*, from mutant analysis and the fine mapping results suggested that TraesCS3D02G542800 encoding gibberellin 2-beta-dioxygenase is the candidate gene of Rht-SN33d [11]. Analyses of the rice brittle culm mutants revealed that *OsBC17* is a novel allele of the *tiller angle control 4 (TAC4)* gene different from previously reported mutants. *OsBC17* regulates rice shoot gravitropism by affecting auxin content and distribution [12]. Another mutant study elucidated the molecular mechanism of maize loose plant architecture 1 (*ZmLPA1*) gene that encodes a zinc-finger protein and regulates maize leaf angle. *ZmLPA1* is involved in auxin biosynthesis and responses [13]. In soybean, *GmRAV* (Related to ABI3/VP1) transcription factor containing both AP2 and B3 domains negatively regulates plant height by directly repressing the gibberellic acid (GA) metabolic gene *GmGA3ox* to reduce GA levels [14].

Grain weight is also a key trait that determines grain yield and is influenced by grain size. The U-box E3 ubiquitin ligase gene *OsPUB43* negatively regulates rice grain size and grain weight by regulating the expression levels of BR-responsive genes and MADS-box genes [15]. Overexpression of a new starch biosynthetic gene of maize (*ZmCBM48-1*), which contains a carbohydrate-binding module 48 (CBM48) domain, altered grain size and thousand grain weight, and increased the starch content in rice [16]. Yoon et al. found that VERNALIZATION INSENSITIVE 3-LIKE 1 (OsVIL1), a homolog of the flowering regulator OsVIL2 in rice, increased grain yield and biomass by directly regulating *OsCKX2* expression [17].

Fatty acid synthesis in seeds plays a crucial role not only in grain germination and seedling morphogenesis but also in human nutrition and grain storage. Yang et al. performed functional studies of two WRINKLED1-Like genes in wheat, *TaWRI1L1* and *TaWRI1L2*, and demonstrated that *TaWRI1L2* is the key transcription factor regulating fatty acid synthesis in bread wheat [18]. Flower color is one of the most important ornamental characteristics of peony tree, in which the yellow petal color is determined by isosalipurposide (ISP). The functional relationship of several metabolic genes (i.e., chalcone synthase gene, *PdCHS*, chalcone isomerase gene, *PdCHI* and chalcone2′-glucosyltransferases gene, *PdTHC2′GT*) with ISP biosynthesis revealed that *PdTHC2′GT* may likely be a critical target for breeding yellow peony tree varieties [19].

Nitrogen transportation and metabolism are critical to plant development. LjAMT2;2, encoding a typical ammonium transporter in *Lotus japonicus*, could be induced during *Arbuscular Mycorrhizal* fungi (AMF) symbiosis, and overexpression of LjAMT2;2 alleviated nitrogen stress and promoted the growth by stimulating ammonium nitrogen uptake [20]. In tea plants, *Lateral Organ Boundaries domain* (*LBD*) transcription factor member, *CsLBD39, acted* as a negative regulator in nitrate signaling and transportation [21]. In Chinese cabbage, *BcSOC1 (SUPPRESSOR OF OVEREXPRESSION OF CONSTANS1)* identified by transcriptome analysis promoted stem elongation, and led to early bolting and flowering [22]. In another work, overexpression of cassava neutral invertase (*Manihot esculenta*) *MeNINV1* in *Arabidopsis* resulted in increased glucose, fructose, and starch contents in leaves with promoted plant growth and delayed flowering time [23].

Environmental stresses represent major adverse factors that threaten food security. TaNBR1, encoding an NBR1-like domain protein in wheat, negatively regulates drought stress response by affecting the expression of drought stress-related genes in *Arabidopsis* [24]. Lysine-specific histone demethylases (*LSDs*) affect gene expression by regulating the methylation level of the H3K4. Liu et al. identified six *LSD-like* (*LDL*) genes from soybean, which are well conserved during soybean domestication and improvement. Most *GmLDLs* are responsive to different abiotic stresses [25]. *GhENODL6*, a member of the early nodulin-like (*ENODL*) family in *Gossypium hirsutum*, plays a role in *Verticillium* wilt resistance by inducing the salicyclic acid (SA) signaling pathway and regulating reactive oxygen species (ROS) production [26]. Bermuda grass (*Cynodon dactylon*) is notoriously resistant to a variety of herbicides. Zheng et al. cloned a cytochrome P450 gene (namely *P450-N-Z1*) from Bermuda grass, and demonstrated that *P450-N-Z1* conferred herbicide-resistance in transgenic soybean plants [27]. Besides the above-mentioned functional studies of genes, the functional characterization of transposons also helped gain new insights. Cong et al. cloned the maize Mutator superfamily members *TED* and *Jittery* and elucidated their structural regions which likely caused the toxic effects on *E. coli* growth [28].

In addition, Hwarari et al. provided a systematic review of the *ICE-CBF-COR* pathway that is key for plants to adapt to cold environments and regulate the cold stress response. They emphasized the cross interconnections of this pathway with other repressors, inhibitors, and activators to induce cold stress acclimation in plants [29].

## 4. Dissecting Important Agronomic Traits through Population Genetics

Genome-wide association study (GWAS) and quantitative trait locus mapping (QTL mapping) have become the major tools to uncover the genetic architectures of complex traits. Sun et al. performed QTL mapping for the two most important traits for soybean production, total flower and pod numbers (TFPN) and effective pod numbers per plant (PNPP), and identified a new locus *qFPN4* controlling both traits [30]. Di et al. carried out a GWAS analysis for 510 Chinese soybean cultivars and identified 37 candidate genes associated with linoleic acid (LA) metabolism in soybean seeds [31].

In wheat, Feng et al. reported a new disomic substitution line containing the *Leymus mollis* (*L. mollis*) 2N chromosomal fragments and, more importantly, identified 13 markers by SLAF-seq for tracing the chromosome 2Ns of *L. mollis* in the wheat line [32]. Another study combined bulked segregant RNA sequencing (BSR-seq), comparative genomics and linkage mapping to map a recessive powdery mildew resistance gene *pmXMM* to the distal end of chromosome 2AL [33]. In maize, Guan et al. mapped four QTLs (qTBN2a, qTBN2b, qTBN4, qTBN6) of the tassel branch number (TBN) trait and identified six candidate genes within the QTL regions. Importantly, one of the candidate genes (*GRMZM2G010011*, *ZmPAT7)*, encoding an S-acyltransferase, was functionally validated by using the CRISPR-Cas9-edited lines [34]. Another maize study also employed QTL mapping to reveal the genetic loci involved in Zn deficiency tolerance with potential candidate genes identified [35]. In rapeseed, a gene determining the multi-inflorescence trait was fine-mapped to a 55-kb interval containing only five genes with *BnaMI* being suggested as the candidate gene [36]. Sodedji et al. used 3120 polymorphic SNPs to identify the QTL regions associated with the carotenoid content in cowpea (*Vigna unguiculata* L. Walp) sprouts and revealed that the carotenoid content is a polygenic trait controlled by genes with additive and dominance effects [37]. A genetic locus controlling the hollow trait in cucumber (*Cucumis sativus* L.) fruit was finely located by BSA-seq and KASP genotyping analysis, suggesting *CsALMT2* as a potential candidate gene [38].

## 5. Multi-Omic Analysis Facilitates Plant Functional Genomic and Genetic Improvement Studies

Comparative transcriptome analysis of two pueraria [*Pueraria lobata* (Willd.) Ohwi] varieties with contrasting resistance to the pseudo-rust revealed the regulatory networks responsive to the pseudo-rust, highlighting that oxidation-reduction process, flavonoid biosynthesis and ABA signaling genes may be associated with the response to pseudo-rust infection [39]. The transcriptome profiling of 42 ginseng (*Panax ginseng*) cultivars suggested that 22 candidate genes involved in ginsenoside Rb1 biosynthesis [40]. By combining time-series transcriptomic profiling with hydrogen cyanide (HCN) analysis of common vetch (*Vicia sativa* L.) seeds [41], Li et al. identified the main regulatory genes for HCN synthesis in common vetch seeds and established a co-expression network of HCN synthesis using the weighted gene coexpression network analysis (WGCNA). The RNA-seq data during cotton fiber elongation between *Gossypium stocksii* (purple fiber) and *Gossypium arboreum* (white fiber) revealed that the key PA biosynthetic genes are responsible for fiber coloration in *G. stocksii*, which improved our understanding in the molecular mechanisms of cotton fiber coloration [42]. Two comparative transcriptomic analyses carried out in *Calotropis gigantea* and zoysiagrass (*Zoysia japonica*), respectively, also helped to obtain insights into the molecular responses to Cd stress and rust resistance, respectively [43,44].

In addition to transcriptomic analysis, multi-omic approaches combining multiple omic datasets are more powerful in gaining insights from a system biology point of view. The integrative omic analysis of mRNA and miRNA expression in cotton revealed some key genes/modules involved in the response to *Verticillium dahliae* stress, providing several candidate genes for further improving *Verticillium wilt* resistance of cotton [45].

Cai et al. revealed the mechanism of anthocyanin biosynthesis in the purple-leaf tea cultivar Zikui (*Camellia sinensis* cv. Zikui) by metabolomics and transcriptomics. Three anthocyanins, petunidin 3-O-glucoside, cyanidin 3-O-galactoside, and cyanidin 3-O-glucoside, were the major anthocyanins in the leaves of Zikui. *CsMYB90* was identified as an important regulator controlling the synthesis of the three anthocyanins [46].

Transcriptomics and metabolomics were used to investigate the underlying mechanisms of chestnut male and female bud transformation in pre- and post-winter. It was found that a higher concentration of GA was beneficial to break the dormancy of flower buds and promoting the development of male flower buds, while a lower concentration of GA and a higher concentration of JA were beneficial to the differentiation and formation of female flower buds in post-winter, in which JAZ1-3 and MYC2-1 might play a key role in female flower bud differentiation [47]. Zhang et al. explored the potential regulatory mechanisms of lignocellulose biosynthesis in *B. napus*, providing a new clue for breeding *B. napus* with lodging resistance in the future [48]. Li et al. reviewed the latest progress in the field of “omics” in sesame and summarized the research progress from the perspective of genome, methylome, transcriptome, proteome and metabolome [49].

Besides the omics and sequencing tools, other molecular techniques showcase their promising use. Kirov et al. sequenced the full-length glutenin genes (*Glu-1Ax*, *Glu-1Bx*, and *Glu-1By*) and their promoters in hexaploid triticale using cas9-targeted nanopore sequencing (nCATS) technology, disclosing the potential of the nCATS approach for sequencing target genes in large genome size plants [50]. A new in vitro gene stacking system, GuanNan Stacking (GNS), was developed by Qin et al. [51]. This system allows multiple expression cassettes to be efficiently assembled in a binary vector simultaneously and is compatible with the Cre enzyme-mediated marker deletion mechanism, emphasizing this technique as a powerful tool for plant metabolic engineering and transgenic breeding of complex traits.

## 6. Concluding Remarks

While the topic covers a wide range of research themes and species, emerging trends have been shown: (1) The genome-wide analysis may be enhanced by comparative analysis in multiple evolutionarily related species to take advantage of the evolution, family expansion/contraction, and expression conservation and divergence between species; (2) Omics data facilitate the narrow down of genetic loci and the underlying candidate genes, thus improving the efficiency of QTL mapping and GWAS studies; (3) Multi-omic analysis could rapidly gain novel insights into a particular biological process, especially in less-studied species. in turn, the identified key candidate genes or regulatory relationships may be functionally characterized in model plants (e.g., Arabidopsis). Utilizing the research trends will certainly strengthen the quality of plant functional genomic studies or genetic improvements. Moreover, application of the cutting edge technologies (for instance, single-cell transcriptomics, spatial transcriptomics and spatial metabolomics) will further push forward crop functional genomics.

## Data Availability

Data is contained within the article.
